# Relationship between running demands in friendly match and aerobic–anaerobic field test results in youth soccer players

**DOI:** 10.1186/s13102-025-01414-w

**Published:** 2025-12-15

**Authors:** Erhan Işıkdemir, Yusuf Köklü, B. Utku Alemdaroğlu, Alper Aşçı

**Affiliations:** 1 Department of Coach Education, Faculty of Sport Science, Nevsehir Hacı Bektas Veli University, Nevşehir, Türkiye; 2 Department of Coach Education, Faculty of Sport Science, Pamukkale University, Denizli, Türkiye; 3 Department of Coach Education, School of Physical Education & Sport, University of Haliç, Istanbul, Türkiye

**Keywords:** Soccer, Aerobic endurance, Anaerobic endurance, Field test, Match performance

## Abstract

**Background:**

Field test performance is a key indicator of soccer match performance, offering insights into aerobic and anaerobic capacities. This study investigated the relationship between field test performance and match performance in young soccer players.

**Methods:**

Thirty-eight elite male soccer players (mean ± SD: age 17.1 ± 1.01 years; height 177.17 ± 5.38 cm; body mass 71.18 ± 5.60 kg) participated in the study. Aerobic endurance was evaluated using the Yo-Yo Intermittent Recovery Level 1 test (YIRT1), the 30–15 Intermittent Fitness Test (30-15_IFT_), and the Circular Field Test (FT_cir_). Anaerobic capacity was assessed through the Repeated Sprint Test and the 40 m Maximal Running Speed Test (MRS_40_). Match performance was determined using GPS data, with metrics including total distance covered and distances in different speed zones: walking (0–6.9 km.h^−1^), low-intensity running (7–12.9 km.h^−1^), moderate-intensity running (13–17.9 km.h^−1^), very high-intensity running (18–20.9 km.h^−1^), sprinting (≥ 21 km.h^−1^), and high-intensity actions (≥ 13 km.h^−1^).

**Results:**

Significant positive correlations were observed between YIRT1 results and total match distance (*r* = .524–.546, *p* < 0.01), as well as high-intensity actions (*r* = .490–.518, *p* < 0.01). The 30-15_IFT_ showed moderately positive correlations with total distance (*r* = .401, *p* < 0.05) and high-intensity actions (*r* = .455, *p* < 0.01). Anaerobic Threshold Running Speed (AnE_RS_) demonstrated a weak to moderate relationship with low-intensity running (*r* = -.397 to .312, *p* < 0.05).

**Conclusions:**

In conclusion, among the tests examined, YIRT1 emerged as the most consistent predictor of match-related running performance. While the 30–15_IFT_ showed a moderate correlation, the relationship with anaerobic tests was limited. These findings highlight the usefulness of YIRT1 in assessing match performance but also reveal that no single test fully reflects the complexity of match performance.

## Introduction

Soccer has a complex structure and involves many short-term movements of low, moderate, and high-intensity [1–4]. During a 90-min match, professional male soccer players cover a total distance of 9.200 to 12.300 m, including 542 to 1168 m of high-intensity running, between 11.2 and 23.2 separate sprints, and 553 to 629 accelerations [5–8]. Therefore, players need well-developed aerobic endurance to sustain performance for the whole duration of the match and to recover quickly between repeated high-intensity efforts [9–11]. They also require anaerobic endurance to execute explosive actions such as sprinting, acceleration, jumping, and changing direction [12–15]. Therefore, it is important to assess players’ aerobic and anaerobic endurance with field or laboratory tests. These evaluations reveal their physical and physiological characteristics and support the design of effective training programs to improve performance.

High performance in aerobic endurance tests is associated with high intensity activities during the match [16]. Therefore, several field tests are used to evaluate aerobic endurance, such as the Yo-Yo Intermittent Recovery Test (YIRT1) [17, 18], the 30–15 Intermittent Fitness Test (30-15_IFT_) [19], and the Circular Field Test (FT_Cir_) [2, 20]. For example, Rebelo et al. [21] and Francini et al. [18] used the YIRT1. They found that soccer players with better performance on this test outperformed their peers regarding total distance, sprint distance, high-intensity activity, and high-intensity running during a match [18, 21]. Meanwhile, Crang et al. [22] used the 30-15_IFT_ on rugby players during the pre-season. They discovered a positive correlation between high-intensity activities during matches and running speeds that exceeded 68% of the 30-15_IFT_ results. They argued that this finding underscores the importance of pre-season training in enhancing capacity for high-intensity effort and highlights the relevance of the 30-15_IFT_ as a performance metric in the sport of rugby. Moreover, Aslan et al. [20] demonstrated a correlation between running speed achieved at lactate concentrations of 2–4 mmol·L^−1^ and the total distance covered during matches. This finding highlights the connection between aerobic capacity and match performance, as well as the role of metabolic responses in shaping running demands during competition. Finally, Altmann et al. [23] reported that players with a higher anaerobic threshold running speed covered greater overall distances, more high-intensity running distances, and sprint distances in matches.

For elite male soccer players, the total amount of time spent engaging in high-intensity activity during a game is around 7 min [24]. Anaerobic energy systems are critical for high-intensity movements such as sprinting, acceleration, deceleration, and changing direction while running [3]. A range of field tests are used for the evaluation of anaerobic endurance, such as the repeated sprint ability test [15] and the 40 m maximal running speed test (MRS_40_) [5]. A number of studies have explored the relationships between such field tests and physical match performance in soccer [6, 15–18, 25]. Rampinini et al. [15] investigated the relationships between RSA scores and match performance, finding that players with better performance on an RSA covered a greater distance at very-high-intensity running and sprinting.

Overall, soccer is a long-duration, intermittent activity that requires high levels of physical endurance to meet the physiological demands of competition, reduce the risk of injury, and optimize post-match recovery. Therefore, the choice of appropriate field tests is crucial to accurately assess players’ physical condition. Young male soccer players can evaluate their aerobic and anaerobic capacity using several of the previously mentioned field tests [6, 14, 16, 21, 26]. Previous studies in adult and professional cohorts have shown associations between field test performance and match locomotor demands, but there is limited evidence for youth players and comparisons of multiple aerobic and anaerobic tests within the same sample. In addition, while friendly matches may not fully reflect competitive demands, they provide a practical context to examine the ecological validity of standardized field tests under full-match conditions. This study aimed to analyze the relationship between aerobic- and anaerobic-based field tests and the total distance covered at different running intensities during friendly matches.

## Methods

### Study design

This study adopted a descriptive correlational design to examine the relationship between field test performance and match performance in elite youth soccer players. Aerobic endurance was measured using the YIRT1, 30-15_IFT_, and FT_cir_, while anaerobic capacity was assessed with the RSA and the MRS_40_. Furthermore, match performance was evaluated during a standardized 90-min friendly game, with total distance, sprint frequency, and high-intensity actions recorded via 10 Hz GPS devices. In addition, all testing procedures and matches were conducted under controlled environmental conditions on a synthetic grass field, thereby ensuring consistency and reliability in data collection.

### Participants

The study sample comprised 40 elite male soccer players (mean ± SD: age 17.1 ± 1.01 years, height 177.2 ± 5.38 cm, body mass 71.2 ± 5.60 kg, training experience 7.0 ± 1.33 years). Participants were required to meet specific eligibility criteria, including being free from lower limb injuries for at least three months prior to the study and having no medical conditions that could hinder their participation. All players were members of the same team and adhered to a structured training regimen of 70 to 90 min per session, six days per week.

The required sample size was calculated using G*Power 3.1 software to ensure statistical adequacy. Based on an effect size of *r* = 0.3, a significance level of α = 0.05, and a statistical power of 1-β = 0.80, the minimum required sample size was 38 participants. To account for possible attrition or incomplete data, an initial sample size of 40 players was recruited. During the data collection phase, two players were excluded due to incomplete GPS data recorded during the competition. Thus, the final analyzed sample consisted of 38 participants, meeting the pre-determined sample size requirement for adequate statistical power. The ethics committee of Nevşehir Hacı Bektaş Veli University approved the study protocol (decision no. 4286, dated 12/02/2019). The study was conducted in accordance with the principles outlined in the Declaration of Helsinki, ensuring respect for the rights and dignity of the participants. Prior to participating in the study, informed written consent was obtained from all participants and, when applicable, their parents.

### Inclusion/exclusion criteria

To ensure the validity and relevance of the study findings, we selected participants based on defined inclusion and exclusion criteria. The inclusion criteria required that young male soccer players aged 16 to 18 have at least five years of soccer training experience and have actively participated in competitive soccer within the previous 12 months. All participants had to be free from injuries or medical conditions that could impair their physical performance. Additionally, players were required to follow a structured training regimen of 70 to 90 min per session, six days a week.

Exclusion criteria included players who failed to provide complete GPS data during match activities, those with extended periods of inactivity due to injury or other reasons, or individuals with medical conditions that could hinder their participation in high-intensity field tests. This study excluded two participants due to incomplete GPS data during the competition.

### Experimental procedure

This study used a descriptive correlational methodology to investigate the relationship between standardized field testing and physical match performance in elite youth soccer players. Aerobic endurance was assessed through three validated field tests: YIRT1, 30-15_IFT_, and FT_cir_. Anaerobic capacity was evaluated using the 6 × 40 m RSA (20 + 20 m) and MRS_40_. Testing protocols were conducted with 48-h recovery intervals to minimize fatigue-related biases and ensure reliability measurements.

Participants followed a standardized nutrition plan prepared by the club dietitian and applied at the training facility. This controlled approach ensured consistent dietary routines and helped standardize physiological conditions during the 15-day evaluation period. To ensure familiarity and minimize learning effects, all participants had previous experience with the testing protocols during their pre-season training. Anthropometric measurements, including height and body mass, were taken on the first day and recorded with high precision (to the nearest 0.005 m for height and 0.1 kg for weight). The testing sessions were structured as follows: the first session included the MRS_40_ and RSA tests, conducted using a high-accuracy electronic timing system. The subsequent sessions featured the YIRT1, 30-15_IFT_, and FT_cir_ tests, administered on separate days with 48-h recovery periods. A friendly match was organized 48 h after the final testing session to evaluate match-specific performance.

The final session consisted of a 90-min friendly soccer match between teams of similar skill levels, conducted by official soccer regulations to ensure standard competitive conditions. Match performance was objectively monitored using a high-resolution 10 Hz GPS tracking system (GPSport EVO; Canberra, Australia; Openfield, Catapult Sports, Melbourne, Australia), which captured detailed data on distance covered, sprint frequency, and movement intensity.

**Fig. 1:**
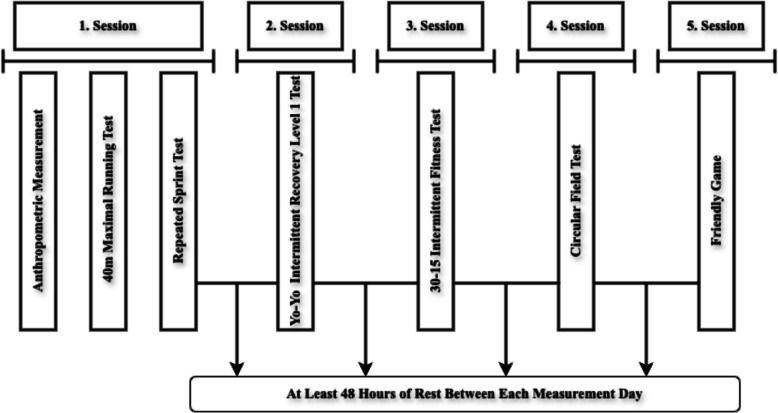
Measurement procedure

All testing procedures and matches were conducted on a standard synthetic grass field routinely used for players’ training, minimizing environmental variability. Tests were carried out consistently (15:00–17:00) under controlled conditions, with air temperatures ranging from 17 °C to 22 °C. During the friendly match, the air temperature was recorded at 18 °C. Players wore sport-specific cleats that complied with competitive soccer standards during all sessions (Fig. 1).

### 40m Maximal Running Speed Test (MRS_40_)

Players started behind the first timing gate and were instructed to sprint as fast as possible over 40 m. All players performed two MRS_40_ tests with 60-s rest intervals, and the better sprint performance was recorded. A wireless electronic timing gate system (Fusion Sport Smartspeed, Australia) was used to measure performance.

### Repeated sprint ability

This test was designed to measure both change of direction and repeated sprint performance. Players completed 6 × 40 m (20 + 20 m) shuttle sprints, each separated by 20 s of passive recovery [15]. The players started from one line, sprinted for 20 m, touched another line with one foot, and returned to the starting line as fast as possible. After the repeated sprint test, the best time in a single trial (RSA_Best_), mean time (RSA_Mean_), and sprint decrement (RSA_Dec_%) were calculated [27]. During the repeated sprint test, a wireless electronic timing gate system was used to record sprint times (Fusion Sport Smartspeed, Australia).

### Yo-Yo Intermittent Recovery Level 1 Test (YIRT1)

The YIRT1 test consists of 20-m shuttle runs at increasing velocities with 10 s of active recovery between them. The YIRT1 starts at 10 km.h^−1^ and then consists of 4 running bouts with increasing speed from 10 to 13 km.h^−1^, seven bouts from 13.5 to 14 km.h^−1^, and after that stepwise 0.5 km.h^−1^ speed increments every eight running bouts. The total distance covered and the final velocity by the player before stopping were then recorded by [28, 29].

### 30–15 Intermittent Fitness Test (30-15_IFT_)

The test comprised 30-s shuttle runs with 15-s recovery intervals. It began at a speed of 8 km·h^−1^ and increased by 0.5 km·h^−1^ after each 45-s stage. The experiment was conducted on a 40-m track, where a pre-recorded beep regulated the speed. During the 15-s recuperation break, players moved forward to the nearest line to commence the subsequent stage. The test concluded when a subject failed, three times in a row, to reach a 3-m zone surrounding each line at the exact moment of the beep signal or due to fatigue. 30-15_IFT_ final velocity (30-15_IFT_ FV) and the running time were recorded after the test lasted for players [19, 30].

### Circular Field Test (FT_Cir_)

The test was performed on the artificial grass pitch, which is a 100 m circular track. The test consists of a 3-min stage at 8, 10, 12, and 14 km.h^−1^ interspersed with 1-min intervals of passive rest [2, 20]. Following each stage, blood samples (5 μL) were obtained from the participants’ earlobes during periods of rest using portable analyzers (Lactate Plus, Nova Biomedical, Massachusetts, USA), while heart rate responses were simultaneously recorded using Polar Team heart rate monitors (Polar Team Sport System, Polar Electro Oy, Finland). These measurements were used to determine each participant’s Anaerobic Threshold Running Speed (AnE_RS_). We used audio beeps from a Conconi-Shuttle Run Timer (Prosport TMR ESC 1100, Tumer Engineering, Ankara, Turkey) to control the running velocity during the test.

### Match performance

The 11-v-11 friendly matches were conducted on a standard-size synthetic grass soccer field, adhering to the same standards as official matches. Two separate matches were organized to include all participants, and each player took part in only one match. Each match consisted of two 45-min halves with a 15-min halftime interval. Matches were played in the late afternoon (16:00), and the teams employed a tactical 1–4-3–3 formation. Before each match, players performed a standardized warm-up routine similar to those used in official games to ensure readiness. Independent referees officiated matches, and all games followed the official rules of soccer.

Match performance variables included total distance covered [31], walking distance (Zone 1: 0–6.9 km.h^−1^), low-intensity running distance (Zone 2: 7–12.9 km.h^−1^), moderate-intensity running distance (Zone 3: 13–17.9 km.h^−1^), high-intensity running distance (Zone 4: 18–20.9 km.h^−1^), sprinting distance (Zone 5: > 21 km.h^−1^), high-intensity activity (HIA: ≥ 13 km.h^−1^), and number of sprints (Sprint_Match_). Additionally, maximum running speed (Max Speed_Match_) [32]. Also, the number of match sprints (Sprint_Match_), maximal running speed (Max Speed_Match_), low- and high-intensity accelerations (Low_Acc_/High_Acc_: > 2.5 m/s^2^), and low- and high-intensity decelerations (Low_Dec_/High_Dec_: < −2.5 m/s^2^) were recorded [18, 33]. Goalkeepers were excluded from the analysis as their physical performance was not monitored. Only players who participated in the full 90 min without substitutions were included in the analysis to ensure data reliability. Substitutions were minimized based on an agreement between the coaches, and players who substituted during the game were excluded from data evaluation. Two players’ data were also excluded due to transfer issues. All GPS data were collected using GPSsport EVO 10 Hz devices (Canberra, Australia; OpenField, Catapult Sports, Melbourne, Australia). These devices ensured accurate and detailed tracking of player movements during the matches.

### Statistical analysis

All statistical analyses were performed using SPSS Statistics 26.0 for Mac (SPSS Inc., Chicago, IL, USA). Results are presented as mean ± standard deviation (SD) and 95% confidence intervals (95% CI). The Shapiro–Wilk test was used to confirm the normal distribution of the data (*n* < 50). Pearson’s product-moment correlation analysis was conducted to determine the relationships between YIRT1, 30-15_IFT_, FT_cir_, RSA, and MRS_40_ performances and their respective associations with match performance variables, including total distance covered, maximal running speed, high-intensity actions, and the number of sprints. Correlation coefficients (r-values) were categorized as follows: < 0.10 trivial, 0.10 to 0.30 small, 0.30 to 0.50 moderate, 0.50 to 0.70 large, 0.70 to 0.90 very large, and 0.90 to 1.00 almost perfect [34]. Additionally, linear regression analysis was employed to assess the explanatory power of independent variables on dependent variables [17]. A significance level of *p* < 0.05 was established for all statistical tests.

## Results

Table 1 presents individual performance values for YIRT1, 30-15_IFT_, FT_cir_, RSA, MRS_40,_ and the time motion analysis for the friendly matches.

**Table 1:** Players’ descriptive and performance characteristics

	**Mean ± *SD***	**Range**
YIRT1 TD (m)	2405.8 ± 566.7	920—3480
YIRT1 FV (km.h^−1^)	17.3 ± 0.9	15.0–19.0
30-15_IFT_ TD (m)	3053.8 ± 377.5	2028—3625
30-15_IFT_ FV (km.h^−1^)	20.6 ± 1.05	17.5—22.0
AnE_RS_ (km.h^−1^)	13.0 ± 1.1	10.8—15.3
RSA_Best_	7.2 ± 0.3	6.5—7.9
RSA_Mean_	7.5 ± 0.2	7.0—8.1
RSA_Decrement_ (%)	5.5 ± 3.1	0.4—14.6
MRS_40_ (km.h^−1^)	29.1 ± 1.0	27.0—31.0
Zone 1 (m)	3619 ± 331	2875–4411
Zone 2 (m)	3464 ± 662.0	1793–4822
Zone 3 (m)	1580 ± 399	1098–2719
Zone 4 (m)	432 ± 107.3	220—762
Zone 5 (m)	433 ± 140	191—756
HIA (m)	2444.4 ± 780	1700—3632
TD (m)	9533 ± 483	7721—1139
Sprint_Match_ (Number)	24.2 ± 6.5	12–41
Max Speed_Match_ (Velocity)	29.3 ± 1.7	25.9–33.3
Low_Acc_ (Number)	110 ± 16.5	84–162
High_Acc_ (Number)	7 ± 1.62	5–12
Low_Dec_ (Number)	118 ± 24.35	72–179
High_Dec_ (Number)	17.4 ± 4.79	8—27

*YIRT1 TD* Yo-Yo Intermittent Recovery Level 1 Test Total Distance, *YIRT1 FV* Yo-Yo Intermittent Recovery Level 1 Test Final Velocity, *30-15*_*IFT*_* TD* 30–15 Intermittent Fitness Test Total Distance, *30-15*_*IFT*_* FV* 30–15 Intermittent Fitness Test Final Velocity, *AnE*_*RS*_ Anaerobic Threshold Running Speed, *RSA*_*TheBest*_ Best time achieved in Repeated Sprint Ability test, *RSA*_*Mean*_ Repeated Sprint Ability test Mean Time, *RSA*_*Devrement*_* %* Repeated Sprint Ability test Sprint Decrement Percentage, *MRS*_*40*_ 40 m Maximal Running Speed Test Mean Velocity, *Zone 1* Mean distance covered at walking Speed, *Zone 2* Mean distance covered at Low-Intensity Running Speed, *Zone 3* Mean distance covered at Moderate Intensity Running Speed, *Zone 4* Mean distance covered at High-Intensity Running Speed, *Zone 5* Mean distance covered at Sprinting Speed, *HIA* High-Intensity Actions (Mean distance covered at Zone3 + Zone4 + Zone5), *TD* Match Total Distance, *Sprint*_*Match*:_Match Sprints, *Max Speed*_*Match*_ Match Maximal Running Speed, *Low*_*Acc*_ Low-Intensity Acceleration, *High*_*Acc*_ High-Intensity Acceleration, *Low*_*Dec*_ Low-Intensity Deceleration, *High*_*Dec*_ High-Intensity Deceleration

Table 2 shows that both YIRT1 and 30-15_IFT_ (TD and FV) performance were moderately to largely associated with match running metrics. Players who achieved greater YIRT1 TD low-to-moderate running zones (Zones 1–4) and accumulated greater match total distance (*R*^2^ = 27%) and high-intensity actions (*R*^2^ = 24%). Similarly, YIRT1 FV demonstrated moderately correlations with distances in Zones 1–4 (*R*^2^ = 15–24%) and largely associations with match TD (*R*^2^ = 29%) and HIA (*R*^2^ = 26%). The 30-15_IFT_ TD is moderately related to Zone 3 (*R*^2^ = 18%), match TD (*R*^2^ = 16%), and HIA (*R*^2^ = 20%). At the same time, the 30-15_IFT_ FV is moderately correlated with Zone 3 (*R*^2^ = 18%), match total distance (*R*^2^ = 16%), and high-intensity actions (*R*^2^ = 20%). In contrast, the anaerobic-threshold running-speed test (AnE_RS_) showed only moderately correlations with low-intensity match zones—Zone 1 (*R*^2^ = 0.15%) and Zone 2 (*R*^2^ = 0.10%), suggesting limited relevance to high-intensity match efforts.

**Table 2:** Pearsons’ correlations (r) between field tests (YIRT1, 30-15_IFT_, AnE_RS_) and match running distance in each Zone

		**YIRT1 TD**	**YIRT1 FV**	**30-15**_**IFT**_** TD**	**30-15**_**IFT**_** FV**	**AnE**_**RS**_
Zone 1	r	-.427^**^	-.444^**^	-.304	-.298	-.397^*^
Zone 2	r	.462^**^	.477^**^	.284	.294	.321^*^
Zone 3	r	.453^**^	.492^**^	.431^**^	.426^**^	.283
Zone 4	r	.387^*^	.392^*^	.274	.272	.312
Zone 5	r	.104	.084	.132	.144	-.055
TD	r	.524^**^	.546^**^	.401^*^	.413^*^	.284
HIA	r	.490^**^	.518^**^	.455^**^	.454^**^	.287

*YIRT1 TD* Yo-Yo Intermittent Recovery Level 1 Test Total Distance, *YIRT1 FV* Yo-Yo Intermittent Recovery Level 1 Test Final Velocity, *30-15*_*IFT*_* TD* 30–15 Intermittent Fitness Test Total Distance, *30-15*_*IFT*_* FV* 30–15 Intermittent Fitness Test Final Velocity, *AnE*_*RS*_ Anaerobic Threshold Running Speed, *Zone 1* Mean distance covered at walking Speed, *Zone 2* Mean distance covered at Low-Intensity Running Speed, *Zone 3* Mean distance covered at Moderate Intensity Running Speed, *Zone 4* Mean distance covered at High-Intensity Running Speed, *Zone 5* Mean distance covered at Sprinting Speed, *HIA* High-Intensity Actions (Mean distance covered at Zone3 + Zone4 + Zone5), *TD* Match Total Distance

^**^p < 0.01. ^*^p < 0.05

Table 3 highlights relationships for sprint-based assessments. Repeated-sprint ability (RSA_Best_) was moderately associated with high-intensity actions (*R*^2^ = 12%), implying that players with faster best sprint times tended to engage in more high-intensity efforts in matches. Conversely, maximal MRS_40_ showed a moderately negative association with high-intensity accelerations (*R*^2^ = 18%), indicating that players with higher top-speed capacity tended to perform fewer short explosive accelerations during match play. No other correlations between sprint tests and match indicators reached significance.

**Table 3:** Pearsons’ correlations (r) between field (RSA and MRS_40_) tests and match performance indicators

		**RST**_**Best**_	**RST**_**Mean**_	**RST**_**Decrement**_	**MRS**_**40**_
Zone 5 (m)	r	-.092	-.086	-.001	-.203
HIA (m)	r	.345^*^	.094	-.220	.286
Sprint_Match_ (Number)	r	-.173	-.118	-.015	-.140
Max Speed_Match_ (Velocity)	r	-.063	-.083	-.145	.053
Low_Acc_ (Number)	r	.041	-.070	-.155	-.042
High_Acc_ (Number)	r	-.201	-.199	.005	-.422^**^
Low_Dec_ (Number)	r	.270	.195	-.133	.301
High_Dec_ (Number)	r	-.227	-.124	.029	-.289

*Zone 5* Mean distance covered at Sprinting Speed, *HIA* High-Intensity Actions (Mean distance covered at Zone3 + Zone4 + Zone5), *Sprint*_*Match*:_Match Sprints, *Max Speed*_*Match*:_ Match Maximal Running Speed, *RSA*_*Mean*_ Repeated Sprint Ability test Mean Time, *RSA*_*Dec*_* %* Repeated Sprint Ability test Sprint Decrement Percentage, *MRS*_*40*_ 40 m Maximal Running Speed Test Mean Velocity, *Low*_*Acc*_ Low-Intensity Acceleration, *High*_*Acc*_ High-Intensity Acceleration, *Low*_*Dec*_ Low-Intensity Deceleration, *High*_*Dec*_ High-Intensity Deceleration

^**^p < 0.01. ^*^p < 0.05

## Discussion

This study aimed to analyze the relationship between aerobic and anaerobic field tests and the total distance covered at different running intensities during official matches. The primary finding of this study suggests that the YIRT1, 30-15I_FT_, FT_Cir_, RSA, and MSR_40_ tests each exhibited varying degrees of correlation with specific physical activities observed during the match. While all tests demonstrated some level of association, the strength of these relationships differed among them. There was a significant correlation between YIRT1 TD and TD covered during the game, and a moderate correlation with 30-15_IFT_ TD. In contrast, no significant correlation was found between anaerobic threshold running speed and HIA or TD covered during the friendly game (Table 2).

The current study also revealed moderate correlations between YIRT1 and the distance covered in all zones, as well as with HIA during the match. This result is consistent with the findings of Castagna et al. [26] who reported a very high correlation between HIA and YIRT1 performance (r: 0.73, *p* < 0.003, 95%CI 0.68–0.83). In another study, Akyıldız and Ocak [35] reported that the YIRT1 TD and the TD covered during a professional soccer match showed a statistically moderate correlation (*r* = 408; *p* = 0.042), but no correlation was observed with other parameters. Taken together, these findings suggest that YIRT1 is a sensitive tool for capturing the aerobic demands associated with overall and high-intensity match running. At the same time, the lack of significant correlations for some variables should not be interpreted as evidence of no relationship, but rather as a possible consequence of limited sample size, player variability, or contextual differences across matches. This highlights the importance of using YIRT1 results as part of a broader monitoring strategy, rather than as a stand-alone predictor of match performance.

This study found a moderate link between 30–15_IFT_ TD and 30–15_IFT_ FV with TD, HIA, and moderate-intensity running during official matches. Crang et al. [22] found that 30-15_IFT_ high-speed running distance was positively correlated with high-speed match activities during rugby matches. Overall, however, our findings indicate that while the 30–15_IFT_ shows moderate associations with certain match variables, it is less effective in predicting running performance at low-intensity, high-intensity, and sprint distances in competitive play. The findings of previous studies on 30-15_IFT_ FV indicate that players’ individual high-intensity training plans can benefit from using 30-15_IFT_ FV [36–38]. At the same time, Buchheit [39] and Thomas et al. [40] emphasized in their study that the 30-15_IFT_ is a valid measurement tool for evaluating aerobic power level. The aerobic system is essential to modern soccer players’ performance because it serves two purposes: helping the anaerobic system recover quickly after bursts of high-intensity activity and providing the main energy source for most of the game. Overall, the findings underline how aerobic and anaerobic pathways interact to shape the physiological responses that determine players’ match performance.

The present study also found that anaerobic threshold running speed had a moderate correlation with the mean low-intensity running distance during the match. However, the TD, HIA, and high-intensity running distance variables displayed during the game showed no relationship with anaerobic threshold running speed. According to Altmann et al. [23], only one of the three seasons showed a significant relationship between the individual anaerobic threshold running speed and the total distance traveled by soccer players during a game. The same study also found no relationship between average running speed, the number of sprints achieved in competitive play, or the individual anaerobic threshold running speed. Aslan et al. [20] conducted a study with 36 young soccer players and found a correlation between the speed at which the anaerobic threshold is reached during running and the total distance covered in the competition. However, there is no correlation between the velocity at which the anaerobic threshold is reached and the distance covered during high-intensity running. Based on these findings, testing the anaerobic threshold running speed is useful in assessing low-intensity running. That being said, increasing an athlete’s anaerobic threshold running speed can improve their overall running distance and their high-intensity running distance performance in competitive events.

This study also found no relationship between the components of the repeated sprint test and the sprint-based movements performed by the players in the match. Similarly, no relationship was found between 40 m maximal sprint components and sprint-based movements in official matches. A study by Rampinini et al. [15], which included 18 elite soccer players, showed a substantial correlation between RSA_MeanTime_ and the total distance covered at high intensity and sprinting speeds during competition. Redvka et al. [6] found a high correlation between distance covered in the Yo-Yo endurance test and the mean number of sprints (*r* = 0.88; *p* < 0.01) in soccer matches. However, the same study found no relationship between %RSA_Dec_ and time-dependent movement analyses during the match. The results of a study conducted with 22 top-level female soccer players by Gonçalves et al. 

[41] revealed that maximal linear sprint bouts had a considerable to very strong relationship with explosive match-play actions, including accelerations, decelerations, and sprint occurrences (*r* = 0.80 to 0.60). However, the same study found no significant relationship between RSA and match performance.

This study’s results clearly show a relationship between physical performance during matches and the aerobic capacity of young male soccer players. This correlation implies that players must improve their aerobic fitness components to cover more distance at high running speeds during matches. These results once again emphasize the importance of aerobic fitness in improving players’ on-field performance. Thus, it is recommended that coaches and sports scientists should be aware of the importance of aerobic fitness components when optimizing players’ physical performance and allocate enough time in their training programs.

### Limitations

This study has several limitations that should be acknowledged. The sample was restricted to two squads (U-17 and U-19) from a single club, which reduces the generalizability of the findings to wider populations of youth soccer players. Moreover, only external load variables were examined; the absence of internal load measures such as heart rate or lactate limits the interpretation of the physiological mechanisms underlying the observed associations. In addition, the cross-sectional design allowed us to identify correlations but not to examine individual adaptations or causal relationships. Another limitation is that match performance was assessed during friendly games, which may not fully reflect the physical and physiological demands of official competitive matches. These factors may have influenced the strength and scope of the results. Future research should therefore employ larger and more diverse samples, integrate both internal and external load indicators, and use longitudinal designs including official competitions to provide a more comprehensive understanding of the relationship between field tests and match performance.

## Conclusions

These findings support the use of YIRT1 and 30-15_IFT_ as practical field tests for evaluating aerobic fitness in young male soccer players, as both showed moderate to strong associations with match-related locomotor activities. In contrast, FT_cir_, MRS40, and RSA demonstrated limited or inconsistent relationships with competitive performance, indicating that they are less effective for predicting match demands. Among all tests, YIRT1 (total distance and final velocity) emerged as the strongest predictor of match performance, emphasizing the central role of aerobic fitness in sustaining high-intensity efforts and facilitating recovery. Coaches and practitioners are therefore encouraged to prioritize YIRT1, alongside 30-15_IFT_, when monitoring players’ fitness and designing training programs. Future studies should build on these findings by including larger and more diverse samples, incorporating both internal and external load measures, and employing longitudinal designs in official competition settings to provide a more comprehensive understanding of the relationship between field tests and match performance.

## Data Availability

The datasets generated and analyzed during the current study are available from the corresponding author on reasonable request.

## Abbreviations

YIRT1: Yo-Yo Intermittent Recovery Level 1 Test
YIRT1 TD: YIRT1 Total Distance
YIRT1 FV: YIRT1 Final Velocity
30-15_IFT_: 30–15 Intermittent Fitness Test
30-15_IFT_ TD: 30-15IFT Total Distance
30-15_IFT_ FV: 30-15IFT Final Velocity
FT_cir_: Circular Field Test
AnE_RS_: Anaerobic Threshold Running Speed
RSA: Repeated Sprint Ability Test
RSA_Best_: Best Sprint Time in RSA
RSA_Mean_: Mean Sprint Time in RSA
RSA_Decrement_ (%): Sprint Decrement Percentage in RSA
MRS_40_: 40 M Maximal Running Speed Test
TD: Total Distance
HIA: High-Intensity Actions (≥ 13 km·h^-1^; sum of Zones 3–5)
Zone 1: Walking distance (0–6.9 km·h^-1^)
Zone 2: Low-intensity running distance (7–12.9 km·h^-1^)
Zone 3: Moderate-intensity running distance (13–17.9 km·h^-1^)
Zone 4: High-intensity running distance (18–20.9 km·h^-1^)
Zone 5: Sprinting distance (≥ 21 km·h^-1^)
Sprint_Match_: Number of sprints during the match
Max Speed_Match_: Maximal running speed achieved during the match
Low_Acc_: Number of low-intensity accelerations (> 2.5 m/s^2^)
High_Acc_: Number of high-intensity accelerations (> 2.5 m/s^2^)
Low_Dec_: Number of low-intensity decelerations (< –2.5 m/s^2^)
High_Dec_: Number of high-intensity decelerations (< –2.5 m/s^2^)
